# Chronic BDE-47 Exposure Aggravates Malignant Phenotypes and Chemoresistance by Activating ERK Through ERα and GPR30 in Endometrial Carcinoma

**DOI:** 10.3389/fonc.2019.01079

**Published:** 2019-10-31

**Authors:** Fan Zhang, Lin Peng, Yiteng Huang, Xueqiong Lin, Li Zhou, Jiongyu Chen

**Affiliations:** ^1^Oncology Research Laboratory, Cancer Hospital of Shantou University Medical College, Shantou, China; ^2^Guangdong Provincial Key Laboratory for Breast Cancer Diagnosis and Treatment, Cancer Hospital of Shantou University Medical College, Shantou, China; ^3^Department of Laboratory Medicine, Cancer Hospital of Shantou University Medical College, Shantou, China; ^4^Health Care Center, The First Affiliated Hospital of Shantou University Medical College, Shantou, China; ^5^Department of Gynecologic Oncology, Cancer Hospital of Shantou University Medical College, Shantou, China

**Keywords:** 2,2′,4,4′-tetrabromo diphenyl ether, endometrial carcinoma, estrogen receptor α, G-protein-coupled receptor-30, cisplatin, paclitaxel, phosphorylated epidermal growth factor receptor, phosphorylated extracellular-regulated protein kinase

## Abstract

Environmental exposure to certain compounds contribute to cell plasticity, tumor progression and even chemoresistance. 2,2′,4,4′-tetrabromo diphenyl ether (BDE-47), one of the most frequently detected polybrominated diphenyl ethers (PBDEs) in environmental and biological samples, is a known estrogen disruptor closely associated with the development of hormone-dependent cancers. However, the effect of BDE-47 on endometrial carcinoma (EC), an estrogen-dependent cancer, remains to be elucidated. Mechanisms of estrogen receptor α (ERα) and G-protein-coupled receptor-30 (GPR30) involved in BDE-47 carcinogenesis are yet to be identified. This study aims to investigate the effect of BDE-47 on the invasive phenotype of estrogen-dependent EC cells. BDE-47-treated cells, such as Ishikawa-BDE-47 and HEC-1B-BDE-47 cells, exhibited increased cell viability and enhanced metastatic ability. *In vivo* studies showed larger tumor volumes and more metastasis in mice injected with Ishikawa-BDE-47 cells compared with parental Ishikawa cells. MTT assay showed that BDE-47 exposure could attenuate sensitivity of EC cells to cisplatin or paclitaxel treatment *in vitro*. Western blotting revealed overexpression of ERα, GPR30, pEGFR (phosphorylated epidermal growth factor receptor), and pERK (phosphorylated extracellular-regulated protein kinase) in Ishikawa-BDE-47 and HEC-1B-BDE-47 cells. Knockdown of ERα or GPR30 by small interfering RNA reversed the stimulating effect of BDE-47 on cell growth, migration and invasion of EC cells. Additionally, treatment with pEGFR or pERK inhibitor impaired cell viability, migration and invasion in Ishikawa-BDE-47 and HEC-1B-BDE-47 cells. Overall, our results indicate that chronic BDE-47 exposure triggers phenotypic plasticity, promotes progression and even chemoresistance in EC cells, at least in part, via ERα/GPR30 and EGFR/ERK signaling pathways.

## Introduction

Endometrial carcinoma (EC) is a major malignant tumor of the female reproductive system (1). The incidence of EC has been increasing in recent decades because of lifestyle change and environmental contamination (2). In China, EC was diagnosed in 63,400 patients and accounted for more than 21,800 deaths in 2016 (3). EC has been proposed to be classified into two pathogenetic groups, of which type I mostly occurs in pre- and peri-menopausal women with a history of unopposed estrogen exposure (4). However, studies found no difference in the positivity of estrogen receptor (ER) or progesterone receptor (PR) as well as the level of sex hormones between these two types of EC. Thus, type II is not completely estrogen-independent (5). Previous studies supported the role of ERα in the development of EC (4, 6). Additionally, G-protein-coupled receptor-30 (GPR30), which encodes a multi-pass membrane protein mediating the estrogen action of intracellular estrogen receptor (7, 8), has been suggested to be an indicator of EC progression (9). However, the etiology and pathogenesis of EC remain poorly defined.

Environmental exposure to certain compounds contributes to cell plasticity and tumor progression (10, 11); an example of these compounds is a group of flame retardants. These synthetic chemical additives are found in consumer products such as building materials, electronics and electrical goods, textiles, and furnishings. Polybrominated diphenyl ethers (PBDEs) are a class of flame retardants most commonly used in a variety of polymers and plastics, as well as in a broad range of consumer products. PBDEs have been produced in notable quantities (12), and consequently, their adverse health effects, environmental persistence and toxicity have raised concerns. Certain kinds of PBDEs (penta-, octa-, and deca-BDE) have been listed as prevalent organic pollutants, and have been banned or voluntarily phased out by manufacturers (13). However, consumer products containing large amounts of PBDEs are still in use and continue to release toxic chemicals into the environment (14). 2,2′,4,4′-tetrabromodiphenyl ether (BDE-47), one of the most predominant PBDE congeners, is characterized by its lipophilic, bio-accumulative, degradation-resistant, and toxic properties. BDE-47 may enter the environment through volatilization and is ultimately found in dust, air, and seafood. It was reported that many products contain BDE-47 and landfill waste containing BDE-47 will persist for many years (15). BDE-47 enters the human body through oral ingestion and inhalation, and can become detectable in various types of human samples including blood, adipose tissue, and human milk (16).

Previous studies about the impact of BDE-47 mostly focused on neurological development, endocrine disruption and tumor initiation. Several studies have revealed that BDE-47 could impair neuronal differentiation (17). BDE-47 could cause DNA damage mediated by oxidative stress, and increase the *in vitro* migration and invasion of human neuroblastoma cells (18–21). Because of the association between PBDEs and hormone levels in humans (22), the impact of PBDEs on hormone-dependent cancers has become a topic of interest. BDE-47 was thought to be an estrogen disruptor with adverse effects on sexual behavior and reproductive function in zebra fish (23). Furthermore, BDE-47 could induce oxidative stress in MCF-7 cells by inhibiting the pentose phosphate pathway (16). An epidemiological survey reported that the serum concentration of BDE-47 in breast cancer women was significantly higher than that of controls (24). However, this pattern was not consistent across all cancers, for instance, BDE-47 could stimulate cell proliferation in human ovarian carcinoma cells OVCAR-3 but not in MCF-7 breast cancer cells (25), reflecting the complicated and inconsistent mechanisms underlying the effect of BDE-47 on different types of cancers.

Chemotherapy is commonly used to treat disseminated or recurrent EC, often after the failure of hormonal therapy. Although the management of EC has undergone a dramatic shift in recent years, and that early-stage EC has a favorable prognosis, the advanced or recurrent EC still has a poor prognosis partially because of chemoresistance. The underlying causes of drug resistance in EC are multi-factorial. Resistance to anti-microtubule agents such as paclitaxel and cisplatin (DDP) is particularly challenging given the importance of these agents in first-line treatment of EC (26). A recent study revealed that cadmium prevented the 5-fluorouracil cytotoxic effect by modifying cell cycle and apoptotic profiles in MCF-7 cells (27). Nonetheless, the potential antagonist effect of BDE-47 against chemotherapy sensitivity of EC has not been well-clarified.

Since EC is an estrogen-dependent cancer and BDE-47 could cause endocrine disruption, we hypothesized that BDE-47 might affect the progression and drug resistance of EC. In this study, the impact of BDE-47 on two human EC cell lines, Ishikawa and HEC-1B cells, was investigated. It has been found that chronic BDE-47 exposure could trigger phenotypic plasticity, promote progression, and even chemoresistance in EC cells, at least in part, via ERα/GPR30 and EGFR (epidermal growth factor receptor)/ERK (extracellular-regulated protein kinase) signaling pathways.

## Materials and Methods

### Cell Lines and Cell Culture

Two endometrial cancer cell lines, Ishikawa (ERα-positive/EGFR-positive), and HEC-1B (ERα-negative/EGFR-positive), were generously provided by Dr. Xiaolong Wei (Cancer Hospital of Shantou University Medical College, Shantou, China) and Dr. Bo Qiu (Southern Medical University, Guangzhou, China). Both these two cell lines have been authenticated. These cells were maintained in complete RPMI 1640 medium (Gibco, ThermoFisher Scientific Inc., California, US), supplemented with 10% fetal bovine serum (FBS, Biological Industry, Kibbutz BeitHaemek, Israel) at 37°C in a 5% CO_2_ incubator. To develop a chronically poisoned cell model, both Ishikawa and HEC-1B cells were exposed to 10 μM BDE-47 (Lot No. 3798900, Chemservice Inc., Worms, Germany) for up to 45 days before the experiments, and were designated as Ishikawa-BDE-47 and HEC-1B-BDE-47, respectively.

### Cell Treatment

To investigate the effect of BDE-47 on paclitaxel- and DDP-induced cytotoxicity in EC cells, Ishikawa-BDE-47 (10 μM), HEC-1B-BDE-47 (10 μM), and their parental cells (1 × 10^4^) were treated with 0, 0.1, 1, 1.25, 5 μM of paclitaxel (Bristol-Myers Squibb Company, New York, USA) and 0, 1.25, 2.5, 5, 10, 20, 50, 100 μM of DDP (Hansoh pharma co. LTD, Jiangsu, China) for 48 h, respectively. After that cell viability was evaluated by MTT assays.

To further identify the cross-talk between ERα/GPR30 and EGFR/ERK signal pathway, 10 μM erlotinib (No. #5083, Cell Signaling Technology Inc., Danvers, Massachusetts, US) and 20 μM PD98059 (No. #9900, Cell Signaling Technology Inc., Danvers, Massachusetts, US) were used to inhibit EGFR autophosphorylation and ERK kinases for 48 h before MTT and Western blotting assay.

### Transfection With siRNA

Twenty-four hours prior to transfection, approximately 8 × 10^4^ cells (Ishikawa-BDE-47 or HEC-1B-BDE-47) were seeded into 6-well plates and grew to 70–80% confluence. These EC cells were then separately transfected with siERα (sc-29305, Santa Cruz Biotechnology, Inc., Dallas, Texas, US) to silence ERα expression, or with siGPR30 (sc-60743, Santa Cruz Biotechnology, Inc., Dallas, Texas, US) to silence GPR30 expression. Control siRNAs (sc-44230, Santa Cruz Biotechnology, Inc., Dallas, Texas, US) was used as the negative control for the parallel experiments. The EC cells were transfected with siRNA oligonucleotides (20 μM) using lipofectamine 3000 (ThermoFisher Scientific Inc., California, US) following manufacturer's protocols, and then collected after 72 h for MTT and Western blotting assay.

### MTT Assay

Cells were separately seeded into 96-well plates at a concentration of 4,000 cells/well, and then subjected to MTT assay. Briefly, MTT assay was performed as follows: cells were first incubated with 5 mg/ml of the sterile filtered MTT solution (Santa Cruz Biotechnology, Inc., Dallas, Texas, US) in phosphate-buffered saline (PBS) and incubated for 4 h in a moist chamber at 37°C, followed by washing with PBS and incubation with DMSO for 10 min with shaking. Solubilized formazan product was detected at OD 490 nm using a microplate reader (Multiskan MK3, ThermoFisher Scientific Inc., California, US). The relative cell viability was calculated using the formula: (OD_treatment_ − OD_blank_)/(OD_control_ − OD_blank_), and the cell inhibition rate was assessed through the formula: 1 − the relative cell viability. Results were summarized by four technical replicates, and each experiment was repeated for triple times.

### Cell Migration and Invasion Assay

The transwell chamber (Lot #5011036, Falcon® Cell Culture Inserts, Krackeler Scientific lnc. Albany, New York, US) was used to detect cell migration and invasion. The bottom of the transwell chamber was made of a polycarbonate membrane with 8 μm membrane pores. For invasion assays, an additional matrigel (50 μl: 50 mg/l, BD biosciences, Franklin Lakes, New Jersey, US) was used to cover the surface of the polycarbonate membrane. Each group of cells (1 × 10^5^ cells suspended in 200 μl serum-free RPMI-1640 medium with 1% BSA) was seeded into the upper chamber, and 600 μl 10% FBS-supplemented RPMI-1640 medium was added to the lower chamber. After 24 h (migration assay) or 48 h (invasion assay), the upper chamber and cells on the upper surface of the membrane were removed. Cells transferred to the lower surface of the membrane were stained with 0.1% crystal violet, and the number of cells was counted under a Leica microscope (Model: DM3000, Wetzlar, Germany). Five fields from each sample were randomly selected for calculating stained cells (28). Results were summarized by three technical replicates, and each experiment was repeated for three times.

### EC Xenografts

Twenty nude mice were purchased from Vital River Laboratory Animal Technology Co. Ltd (Beijing, China). The animal protocol was reviewed and approved by the Medical Animal Care and Welfare Committee at Shantou University Medical College. The body weights of the mice ranged from 20 to 25 g. The mice were randomly allocated into two groups. The right axilla of ten mice was subcutaneously injected with 4 × 10^6^ Ishikawa or Ishikawa-BDE-47 (10 μM) cells. The growth of xenograft tumors was measured every 3 days using a digital caliper, and tumor volume was calculated using the formula: Length × Width^2^ ×0.5. After 31 days, mice were euthanized, and the xenograft tumors were dissected. Additionally, other ten mice were used to detect effect of BDE-47 on the migration ability of EC cells. In that case, 5 ×10^6^ Ishikawa or Ishikawa-BDE-47 (10 μM) cells were given intravenously through the tail. After 14 days, mice were euthanized, and potential metastasis were found and counted in the cervical, axillary, and abdominal lymph nodes, lung and liver. Lymph nodes were fixed and embedded in paraffin for histology and immunohistochemistry (IHC) staining analyses.

### Hematoxylin-Eosin Staining and Immunohistochemistry

Lymph nodes of mice were collected, fixed in 10% phosphate-buffered formalin for 24 h, and embedded in paraffin wax. Sections of tumors (4 μm in thickness) were stained with hematoxylin-eosin and pictured under a light microscope (DX45, Olympus Microsystems Ltd., Japan). IHC for Pan Cytokeratin (Kit-0009, MXB Biotechnologies, Fuzhou, China) was carried out on sections using a standard EnVision complex method. After deparaffinization and rehydration, endogenous peroxidase activity was blocked with 0.3% hydrogen peroxide for 30 min. Then tissue sections were autoclaved at 121°C in citrate buffer (pH 6.0) for 10 min, and incubated with mouse anti-Cytokeratin monoclonal antibody. IHC staining was carried out with the anti-Mouse/Rabbit (Kit-5030, MXB Biotechnologies, Fuzhou, China) and 3,3′-diaminobenzidine as the chromogen substrate. Negative control was obtained by replacing the primary antibody with normal rabbit IgG.

### Western Blotting (WB)

For WB analysis, cells were first lysed with a cell lysis buffer containing PMSF (Phenylmethanesulfonyl fluoride, both from Beyotime, Shanghai, China) on ice for 30 min and centrifuged at 12,000 rpm for 15 min at 4°C to remove cell debris. Proteins (50 μg) of each cell lysate were then separated by SDS-PAGE and transferred onto a PVDF membrane followed by blocking with Tris-buffered saline containing 0.05% Tween 20 (TBST) and 5% non-fat milk for 1 h at room temperature, washed 3 times for 5 min each in TBST, and incubated at 4°C overnight with either mouse anti-GAPDH monoclonal antibody (1:3,000, ZSGB-BIO, Beijing, China), rabbit anti-phospho-EGF receptor (Tyr1148) polyclonal antibody (EGFR antibody, 1:1,000, #4404, Cell Signaling Technology Inc., Danvers, Massachusetts, US), rabbit anti-GPR30 polyclonal antibody (1:1,000, sc-48525-R, Santa Cruz Biotechnology Inc., Dallas, Texas, US), rabbit ERα monoclonal antibody (1:1,000, #2294327, Millipore Inc., Darmstadt, Germany), rabbit P-EGFR antibody (Y1148) (pEGFR antibody, 1:1,000, #4404S, Cell Signaling Technology Inc., Danvers, Massachusetts, US), rabbit P-p44/42 MAPK antibody (T202/Y204) (pERK antibody, 1:1,000, #4370S, Cell Signaling Technology Inc., Danvers, Massachusetts, US), or rabbit p44/42 MAPK antibody (ERK antibody, 1:1,000, #4695S, Cell Signaling Technology Inc., Danvers, Massachusetts, US) in blocking buffer. Following washes with TBST (3 times for 5 min each), the blots were incubated with horseradish peroxidase-labeled anti-rabbit (1:5,000, Novus Biologicals, Littleton, Massachusetts, US) or anti-mouse (1:3,000, Santa Cruz Biotechnology Inc., Dallas, Texas, US) IgG at room temperature for 2 h, washed with TBST, and observed through chemiluminescence (ChemiDoc^TM^ XRS+, Bio-Rad Laboratories, Inc., Hercules, California, US).

### Statistical Analysis

Statistical analyses were performed using the SPSS 13.0 software package (SPSS Inc., Chicago, IL). The comparisons of cell viability, cell numbers representing the capacity of cell migration and invasion, and xenograft tumor size between different treatment groups were conducted using *t*-tests. For all tests, a *P*-value of < 0.05 was considered significant.

## Results

### BDE-47 Boosted Viability and Metastatic Capacity of EC Cells

**Figure 1:**
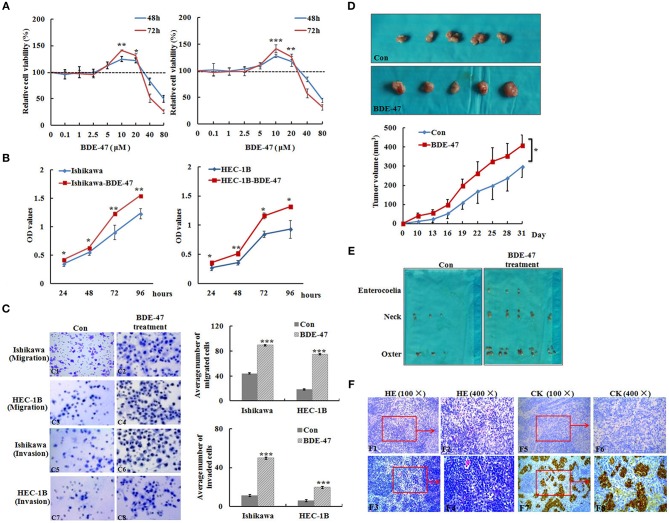
Effect of BDE-47 on the viability and metastasis of EC cells. **(A)** MTT assay on Ishikawa (Left panel) and HEC-1B (Right panel) cells treated with BDE-47 at the concentrations of 0.1, 1, 2.5, 5, 10, 20, 40, and 80 μM for 48 and 72 h. **(B)** OD values reflecting the viability capacity of Ishikawa (Left panel) and HEC-1B cells (Right panel) recorded at 24, 48, 72, and 96 h after cells were exposed to BDE-47 at 10 μM for 45 days. **(C)** Cell migration (C1–C4) and invasion (C5–C8) assay on Ishikawa/Ishikawa-BDE-47 and HEC-1B/HEC-1B-BDE-47 cells. Representative images (400×) of cell migration (C1–C4) and invasion (C5–C8) are shown on the left side. **(D)** Primary xenograft tumors of Ishikawa cells and tumor volumes. **(E)** Lymph nodes containing potential metastasis from mice were found and shown. **(F)** Upper panel: HE staining assessed cellular morphology of lymph nodes with or without metastatic tumors. F1: 100×, F2: 400×, F3: 100×, F4: 400×). Lower panel: IHC staining of cytokeratin negative in F5 (100×) and F6 (400×), and positive in F7 (100×) and F8 (400×). CK, Cytokeratin; Con, Ishikawa cells without BDE-47 treatment. *P < 0.05; **P < 0.01; ***P < 0.001.

Ishikawa and HEC-1B cells were incubated with different concentrations (0.1, 1, 2.5, 5, 10, 20, 40, and 80 μM) of BDE-47. The positive effect of BDE-47 on cell viability was observed at concentrations of 10 μM (relative cell viability at 48 and 72 h after BDE-47 treatment: 1.246, *P* = 0.005; 1.416, *P* < 0.001) and 20 μM (1.218, *P* = 0.014; 1.319, *P* < 0.001) in Ishikawa cells, as well as in HEC-1B cells at concentrations of 10 μM (1.282, *P* < 0.001; 1.416, *P* < 0.001) and 20 μM (1.179, *P* < 0.001; 1.265, *P* = 0.001) in contrast to that in their parental cells (Figure 1A). Based on these data, 10 μM was selected as the chronic BDE-47 treatment concentration in Ishikawa and HEC-1B cells. MTT assays showed that the cell viability of Ishikawa-BDE-47 cells was slightly increased in contrast to Ishikawa cells after 24 h (OD values: 0.42 vs. 0.35, *P* = 0.045) and 48 h (0.63 vs. 0.55, *P* = 0.031), and the increase was more obvious after 72 h (1.23 vs. 0.90, *P* = 0.008) and 96 h (1.55 vs. 1.23, *P* = 0.001). A similar trend was observed in HEC-1B-BDE-47 cells and their parental cell line, of which OD values were 0.36 and 0.27 at 24 h (*P* = 0.010), 0.51 and 0.36 at 48 h (*P* = 0.001), 1.16 and 0.84 at 72 h (*P* = 0.019), and 1.32 and 0.93 at 96 h (*P* = 0.013; Figure 1B).

To examine whether BDE-47 could promote metastasis in EC cells, transwell chamber assays were performed. As shown in Figure 1C, the average number of Ishikawa-BDE-47 cells migrating across the membrane was significantly higher than that of the parental cells (89.6 ± 8.0 vs. 44.0 ± 8.1, *P* < 0.001). Similarly, the average number of migrated HEC-1B-BDE-47 cells was evidently higher than that of HEC-1B cells (75.0 ± 13.7 vs. 18.4 ± 5.8, *P* < 0.001). Moreover, both Ishikawa-BDE-47 (number of invaded cells: 49.8 ± 8.8 vs. 11.2 ± 0.45, *P* < 0.001) and HEC-1B-BDE-47 (19.6 ± 2.5 vs. 6.0 ± 1.3, *P* < 0.001) showed more invasive ability as compared to their parental cells. These results indicated that Ishikawa-BDE-47 and HEC-1B-BDE-47 cells have increased the cell viability and metastatic capacity.

### BDE-47 Enhanced the Growth and Metastasis of EC *in vivo*

In order to understand the effect of BDE-47 on EC cell growth *in vivo*, Ishikawa cells were subcutaneously injected into mice. As shown in Figure 1D, tumor volumes of Ishikawa xenografts on days 10, 22, and 31 reached 11.08 mm^3^ (±4.38), 167.11 mm^3^ (±37.46), and 296.78 (±65.71) mm^3^, respectively, while larger tumor sizes were observed in Ishikawa-BDE-47 xenografts: 39.85 mm^3^ (±14.83, *P* = 0.010), 261.76 mm^3^ (±61.91, *P* = 0.040), and 408.95 mm^3^ (±54.83, *P* = 0.040). Additionally, potential metastasis were found and quantified. In the mice inoculated with Ishikawa-BDE-47 cells, there were 22, 10, and 4 metastatic tumors confirmed by cytokeratin IHC staining (Figures 1E,F, Table 1) in the lymph nodes of the neck, axilla and enterocoele of these mice, respectively. However, only one metastasis in the lymph nodes of the neck was found in the mice with Ishikawa xenografts (Figures 1E,F, Table 1). The cellular morphology of metastasis was assessed by HE staining (Figure 1F).

**Table 1:** Summary of the numbers of lymph nodes with or without metastatic tumors by Cytokeratin IHC staining.

**Characteristics**	**Control**	**BDE-47**
Number of mice	5	5
Total lymph nodes^a^	8	37
Without met^b^	7	1
With met^c^	1	36
Proportion (%)^d^	12.5	97.3
**NECK**		
Total lymph nodes^e^	4	22
Without met^f^	3	0
With met^g^	1	22
Proportion (%)^h^	25	100
**AXILLA**		
Total lymph nodes^e^	4	10
Without met^f^	4	0
With met^g^	0	10
Proportion (%)^h^	0	100
**ENTEROCOELE**		
Total lymph nodes^e^	0	5
Without met^f^	0	1
With met^g^	0	4
Proportion (%)^h^	0	80

a *Total number of extracted lymph nodes*.

b *Lymph nodes without metastasis*.

c *Lymph nodes with metastasis*.

d *The proportion of lymph nodes with metastasis to all extracted lymph nodes*.

e *Total number of extracted lymph nodes from neck, axilla, or enterocoele*.

f *Lymph nodes without metastasis from neck, axilla, or enterocoele*.

g *Lymph nodes with metastasis from neck, axilla, or enterocoele*.

h *The proportion of lymph nodes with metastasis to all extracted lymph nodes from neck, axilla, or enterocoele*.

### BDE-47 Lessened the Paclitaxel and DDP Cytotoxic Effects on EC Cells

**Figure 2:**
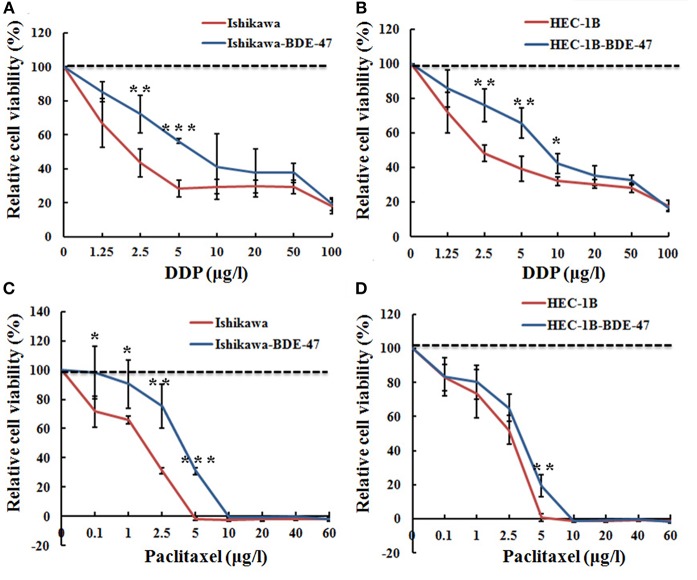
Relative cell viability of Ishikawa-BDE-47, HEC-1B-BDE-47 and their parental cells before and after treatment with DDP or paclitaxel. MTT assays were performed before and after retreatment with different concentrations of DDP for 48 h in Ishikawa-BDE-47 **(A)**, or in HEC-1B-BDE-47 cells **(B)**, as well as their parental cells. Different concentrations of paclitaxel were also used to treat Ishikawa and Ishikawa-BDE-47 **(C)**, as well as HEC-1B and HEC-1B-BDE-47 **(D)**, and then after 48 h, relative cell viability was assessed. *P < 0.05; **P < 0.01; ***P < 0.001.

We next explored whether BDE-47 exposure could attenuate sensitivity of EC cells to DDP or paclitaxel. As shown in Figures 2A,B, both of Ishikawa-BDE-47 and HEC-1B-BDE-47 cells consistently exhibited lower DDP sensitivity compared to their parental non-treated cells at different concentrations. The cell inhibition rates of Ishikawa-BDE-47 at the concentration of 2.5 and 5 μg/l were 0.275 and 0.436, while these numbers of Ishikawa cells were 0.555 and 0.711 (*P* = 0.007; *P* < 0.001). Similarly, the cell inhibition rates in HEC-1B at the concentrations of 2.5, 5, and 10 μg/l were 0.517, 0.609, and 0.679, but separately reduced to 0.234, 0.337, and 0.573 in HEC-1B-BDE47 cells (*P* = 0.002; *P* = 0.003; *P* = 0.014). Regarding paclitaxel, BDE-47 treatment also reduced its cytotoxicity to Ishikawa and HEC-1B cells. As seen in Figure 2C, Ishikawa-BDE-47 had significantly lower cell inhibition rates than that in Ishikawa cells, when exposed to paclitaxel (at 0.1 μg/l: 0.088 vs. 0.341, *P* = 0.029; 1 μg/l: 0.158 vs. 0.339, *P* < 0.001; 2.5 μg/l: 0.332 vs. 0.688, *P* < 0.001; 5 μg/l: 0.689 vs. 1.018, *P* < 0.001). Likewise, HEC-1B-BDE-47 displayed an inferior cell inhibition rate with the treatment of paclitaxel at the concentration of 5 μg/l compared to its parental cells (0.809 vs. 0.993, *P* = 0.002, Figure 2D).

### BDE-47 Increased Viability and Metastatic Capacity of EC Cells Through the ERα/GPR30 and ERFR/ERK Signaling Pathways

**Figure 3:**
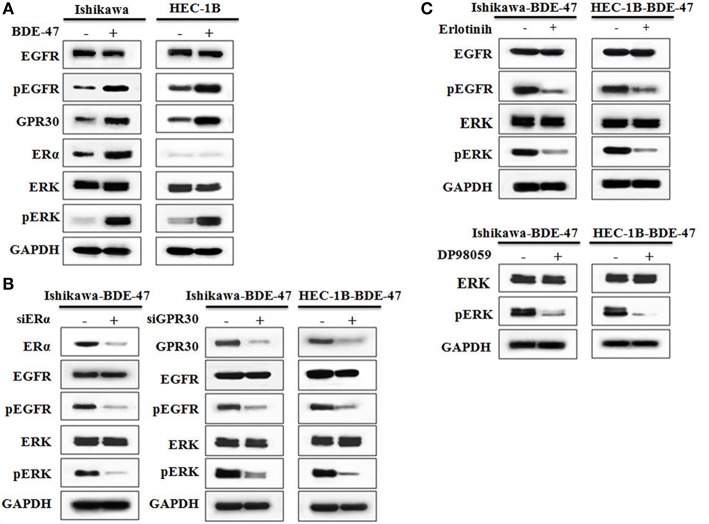
Western blotting on protein expressions of ERα, GPR30, EGFR, pEGFR, ERK, and pERK. **(A)** Protein expression in Ishikawa and HEC-1B cells with or without exposure to 10 μM BDE-47. **(B)** Protein expression in Ishikawa-BDE-47 and HEC-1B-BDE-47 cells treated with or without siERα, and siGPR30. **(C)** Expression of pEGFR and EGFR in Ishikawa-BDE-47 and HEC-1B-BDE-47 after pEGFR inhibitor (erlotinib) treatment (left). Expression of pERK and total ERK in Ishikawa-BDE-47 and HEC-1B-BDE-47 after pERK inhibitor (DP98059) treatment (right).

Estrogens are steroid hormones that regulate a plethora of physiological processes in mammals. Activation of ERα by estrogens is a well-known mechanism underlying EC development. Expression of GPR30 has been suggested to be a novel indicator of EC progression (9). As shown in Figure 3A, levels of both ERα and GPR30 proteins were elevated in ERα-positive Ishikawa-BDE-47 cells when compared to that in their parental cell line. In ERα-negative HEC-1B cells, BDE-47 treatment significantly up-regulated GPR30 expression. Since activation of EGFR/ERK signaling pathway through ERα/GPR30 was present in other cancer cells (29), we detected proteins of EGFR, pEGFR, ERK, and pERK by Western blotting. Significant up-regulation of pEGFR and pERK, but not total EGFR and ERK expression, was seen in Ishikawa-BDE-47 cells compared to that in Ishikawa cells. A similar phenomenon was observed in HEC-1B-BDE-47 and its parental cells. These results indicated a cross talk between ERα/GPR30 and ERFR/ERK signaling pathways potentially underlying the increased proliferative and metastatic effect of BDE-47.

### Down-Regulation of ERα/GPR30 Attenuated Viability and Metastatic Capacity of EC Cells Induced by BDE-47

**Figure 4:**
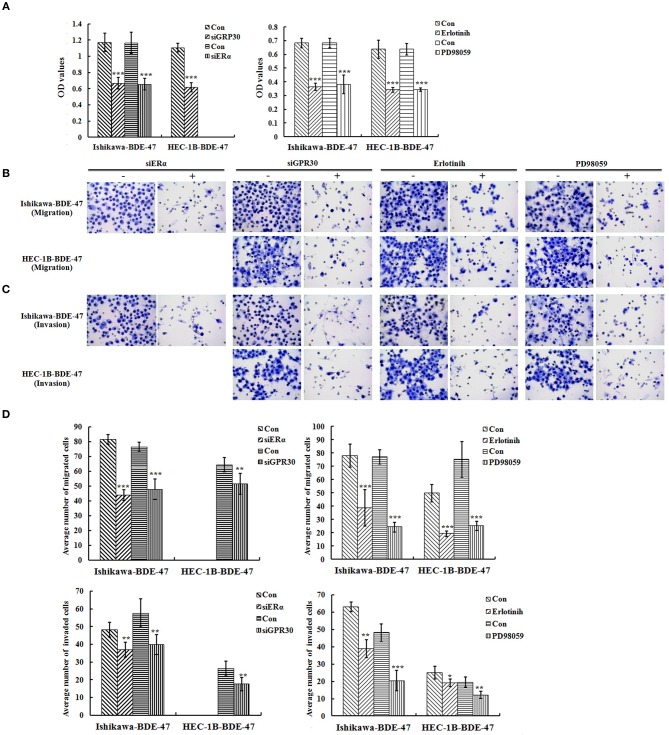
Role of ERα/GPR30 and EGFR/ERK signaling pathway underlying the viability and metastatic effect induced by BDE-47. **(A)** Cell viability of Ishikawa-BDE-47 and HEC-1B-BDE-47 cells at 72 h after transfection with siERα or siGPR30, or the treatment with pEGFR inhibitor (erlotinib) or pERK inhibitor (PD98059) for 48 h. **(B–D)** The migration and invasive capacity of Ishikawa-BDE-47 and HEC-1B-BDE-47 cells after transfection with siERα or siGPR30, and treatment with pEGFR inhibitor (erlotinib) or pERK inhibitor (PD98059). Representative images (400×) of cell migration **(B)** and invasion **(C)** are shown. Con: refer to Ishikawa-BDE-47 or HEC-1B-BDE-47 without any other treatment. *P < 0.05; **P < 0.01; ***P < 0.001.

To investigate the role of ERα/GPR30 in BDE-47 induced EC cell viability, Ishikawa-BDE-47 (ERα+/GPR30+) cells were treated with siERα and siGPR30. Because of its ER-negativity, HEC-1B-BDE-47 cells were only treated with siGPR30. As shown in Figure 3B, significant reductions in ERα and GPR30 expression were observed in Ishikawa-BDE-47-siER and Ishikawa-BDE-47-siGPR30 cells in contrast to that in Ishikawa-BDE-47 cells. Similarly, GPR30 expression was reduced in HEC-1B-BDE-47-siGPR30 compared to that in HEC-1B-BDE-47 cells. MTT assay also revealed significant inhibition of cell viability in Ishikawa-BDE-47-siER cells (OD values at 24, 48, 72 h: 0.42 vs. 0.40, *P* = 0.500; 0.63 vs. 0.48, *P* = 0.005; 1.17 vs. 0.66, *P* < 0.001) and Ishikawa-BDE-47-siGPR30 cells (OD values at 24, 48, 72 h: 0.46 vs. 0.39, *P* = 0.296; 0.65 vs. 0.46, *P* = 0.008; 1.17 vs. 0.67, *P* < 0.001) when compared to their parental cells. Similarly, a significant reduction in cell viability was seen in HEC-1B-BDE-47-siGPR30 in contrast to HEC-1B-BDE-47 (OD values at 24, 48, 72 h: 0.44 vs. 0.39, *P* = 0.367; 0.63 vs. 0.48, *P* = 0.012; 1.11 vs. 0.62, *P* < 0.001). Figure 4A illustrates the MTT results of siRNA at 72 h. Additionally, the transwell assay revealed an impaired capacity of metastasis in Ishikawa-BDE-47-siER (number of migrated cells: 44.0 vs. 81.4, *P* < 0.001; number of invaded cells: 37.0 vs. 48.2, *P* = 0.003, Figures 4B–D) and Ishikawa-BDE-47-siGPR30 (number of migrated cells: 47.8 vs. 76.4, *P* < 0.001; number of invaded cells: 39.8 vs. 57.6, *P* = 0.003, Figures 4B–D) compared to the corresponding parental cells. A similar trend was seen in HEC-1B-BDE-47-siGPR30 and its parental cells (number of migrated cells: 51.6 vs. 64.4, *P* = 0.010; number of invaded cells: 17.6 vs. 26.2, *P* = 0.009, Figures 4B–D).

### Inhibition of pEGFR or pERK Weakened Viability and Metastatic Capacity in BDE-47-Treated EC Cells

As shown in Figure 3B, gene knock-down using siGPR30 or siER down-regulated the expression of pERK and pEGFR but not total ERK and EGFR. To further demonstrate the role of EGFR/ERK signaling pathway in EC cell viability induced by BDE-47, a pEGFR inhibitor or a pERK inhibitor was applied to Ishikawa-BDE-47 and HEC-1B-BDE-47 cells (Figure 3C). As shown in Figure 4A, the pEGFR inhibitor significantly down-regulated the extent of cell viability and metastasis stimulated by BDE-47 in both Ishikawa-BDE-47 and HEC-1B-BDE-47 cells. OD values at 48 h of Ishikawa-BDE-47 treated with and without the pEGFR inhibitor at 10 μM were 0.36 and 0.68 (*P* < 0.001), indicating significant inhibition. Similarly, notable suppression was seen in HEC-1B-BDE-47 cells treated with the pEGFR inhibitor at a concentration of 10 μM (OD values at 48 h: 0.34 vs. 0.64, *P* < 0.001). The EGFR/ERK signaling pathway is the classical MAPK pathway. The pERK inhibitor seemed to block the effect of BDE-47 on Ishikawa and HEC-1B cells. As shown in Figure 4A, OD values at 48 h of Ishikawa-BDE-47 was 0.68, but this was reduced to 0.38 (*P* < 0.001) after the pERK inhibitor treatment at a concentration of 20 μM. Similarly, OD value of HEC-1B was reduced from 0.64 to 0.34 (*P* < 0.001) after the pERK inhibitor treatment. These results suggested that the EGFR/ERK signaling pathway might be at the downstream of ER/GPR30 and be involved in BDE-47-induced EC cell viability.

The transwell assay was used to examine the metastatic capacity of Ishikawa-BDE-47 and HEC-1B-BDE-47 cells treated with a pEGFR inhibitor at 10 μM or a pERK inhibitor at 20 μM for 48 h. After the addition of a pEGFR inhibitor, the number of migrated and invaded cells was reduced from 78.0 ± 8.8 to 38.8 ± 13.7 (*P* < 0.001), and from 63.0 ± 2.7 to 38.8 ± 5.2 (*P* < 0.001, Figures 4B–D), respectively. The same trend of cell invasion and migration was also seen in HEC-1B-BDE47 cells after the pEGFR inhibitor treatment. The number of migrated cells was reduced from 49.8 ± 6.5 to 19.2 ± 2.2 (*P* < 0.001), and the number of invaded cells was reduced from 25.0 ± 3.6 to 19.2 ± 2.2 (*P* = 0.015). Likewise, as shown in Figures 4B–D, treatment with a pERK inhibitor for 48 h impaired the metastatic ability of Ishikawa-BDE-47 and HEC-1B-BDE-47 cells, accompanied with reductions in the numbers of migrated and invaded cells from 77.0 ± 5.43 to 24.2 ± 3.77 (*P* < 0.001, Figure 4D left panel for Ishikawa-BDE-47), from 48.2 ± 4.92 to 20.4 ± 5.86 (*P* < 0.001, Figure 4D right panel for Ishikawa-BDE-47), from 75.0 ± 13.69 to 25.2 ± 3.49 (*P* < 0.001, Figure 4D left panel for HEC-1B-BDE-47), and from 19.6 ± 2.88 to 12.2 ± 2.05 (*P* = 0.001, Figure 4D right panel for HEC-1B-BDE-47).

## Discussion

PBDEs are considered as endocrine-disrupting chemicals with thyroxine- and estrogen-like effects (30). One publication by Li et al. revealed that PBDE-209 increased the viability and proliferation of cells in several types of cancer, including breast cancer, cervical cancer, and ovarian cancer. Interestingly, PBDE-209-up-regulated phosphorylation of ERK was also observed (31). In colon cancer HCT-116 cells, BDE-99 was found to increase the cell migration and invasion as well as to trigger EMT (epithelial–mesenchymal transition), most likely via the PI3K/AKT/Snail signaling pathway (11).

Interestingly, BDE-47 could exert a differential effect on different types of cancer cells. In human neuroblastoma SH-SY5Y cells, BDE-47 had limited cytotoxicity but significantly increased the *in vitro* cell migration and invasion by up-regulating MMP-9 through the GPR30/PI3K/Akt signaling pathway (21). In OVCAR-3cells, BDE-47 was found to stimulate cell proliferation by activating CDK1, CDK7, E2F1, and E2F2; however, this effect was not observed in MCF-7 cells. Notably, BDE-47 had no effect on ERα protein expression in OVCAR-3 cells, while decreased ERα protein expression in MCF-7 cells. Additionally, BDE-47 had no effect on ERK phosphorylation in the two cell lines (25). In contrast, Kanaya et al. recently reported that BDE-47 stimulated proliferation of an estrogen-dependent breast cancer cell line MCF-7aroERE and induced ER-regulated genes expression, which suggested that BDE-47 acted as a weak agonist of both ERα and estrogen-related receptor α (ERRα) (32). One previous case-control study from our laboratory demonstrated that BDE-47 level was positively with breast-cancer risk regardless of ER stratification (33). Similarly in this study, BDE-47 treatment enhanced the cell growth, invasion and migration in both Ishikawa and HEC-1B cells.

The differences between the above studies may attribute to cell specificity, exposure time, and moreover, a different effect for the parent compound and its metabolites. We noticed that the exposure model in Karpeta's research was short-term treatment for 72 h, while that in Kanaya's paper and the present study were longer for 5 days and up to 45 days, respectively. Furthermore, hydroxylated metabolites of PBDE were found to be more potent agonists of estrogen receptors than the parent compounds (34). For instance, BDE-47 had no effect on ERα and ERβ protein expression in OVCAR-3 cells, whereas 5-OHBDE-47 upregulated ERα only and 6-OH-BDE-47 increased both ERα and ERβ protein expression (25). Previous studies have indicated that CYP2B6, a predominant cytochrome P450s, was involved in the formation of hydroxylated PBDEs (OH-PBDEs) (35). Although no evidence has been established for the expression of this enzyme in EC cells, detecting hydroxylated BDE-47 in the culture medium of Ishikawa-BDE-47 and HEC-1B-BDE-47 cells will help to exclude the potential effect of hydroxylated metabolites of BDE-47 on EC cell biology in the present study.

ERα, a ligand-activated transcription factor localizing in the nucleus, is expressed in approximately 80–90% of endometrioid tumors. The estrogen-ERα signaling pathway is implicated in increased uterine growth (36). The newly discovered membrane estrogen receptor GPR30, expressed in ~80% of endometrioid tumors (5), is a specific receptor for 17β-estradiol involved in the non-genomic effect of estrogen. GPR30 was also addressed to mediate the proliferative and invasive effects of estrogen and tumorigenesis in EC cell line (37) and suggested to be an indicator of clinical outcomes of EC (9). It is well-known that the classical mechanism of estrogen action involves binding to the ERα and ERβ, which regulates transcription through direct binding at estrogen-responsive elements in the promoter region of target genes or through tethering to other well-known transcription factors such as AP1, SP1, and nuclear factor κB (38). Emerging evidence has indicated that GPR30 mediated invasion and carcinogenesis induced by 17-β estradiol in an EC cell line (37). To highlight a possible estrogen receptors involvement in the effect of BDE-47 on EC cells, we detected the status of ERα and GPR30 in these two cell lines. The upregulation of ERα and GPR30 in Ishikawa-BDE-47 cells and increased GPR30 expression in HEC-1B-BDE-47 suggest that both ERα and GPR30 are the main targets of BDE-47. Furthermore, siRNA for ERα or GPR30 in BDE-47-treated Ishikawa and HEC-1B cells attenuated the catalytic role of BDE-47 on malignant phenotypes. Since the upregulation of ERα and GPR30 in BDE-47-treated EC cells, we speculate BDE-47 carried out its cancer-promoting effect on EC cells via estrogen molecular pathways. Mitogen-activated protein kinase (MAPK) cascades are key signaling pathways involved in various cellular functions, including cell proliferation, differentiation, survival, and metastasis. EGFR/ERK is the classical MAPK signaling pathway. Recently, a link between environmental chemicals such as Bisphenol A and cancer progression has been suggested in ER-negative in flammatory breast cancer cells, in which EGFR/ERK signaling was involved (39). It is believed that the cross-talk between EGFR and ERα plays a critical role in the regulation of breast cancer development (40). Polychlorinated biphenyl 104 was found to promote migration and invasion of endometrial stromal cells by inducing the expression of MMP-3 and MMP-10, which may involve in the EGFR signaling pathway (41). The cross talk between GPR30 and EGFR/ERK signaling pathways is supported by other studies. GPR30-EGFR signaling pathway was reported to be involved in Bisphenol A promoting the proliferation of leiomyoma cells (42). One study from Zhang et al. (43) revealed that tamoxifen, known for its agonist activity on GPR30, could up-regulate the phosphorylated protein expression of both EGFR and ERK in EC cells, without altering total EGFR/ERK protein expression, indicating that the growth stimulation of EC cells in response to 4-OH tamoxifen was via the GPR30/EGFR pathway. Another publication noted that both estradiol and tamoxifen could induce cell migration through GPR30 in EC cells with or without ERα expression, accompanied by elevated ratios of pEGFR/Total-EGFR and pERK/Total-ERK. ERα could only be partially involved in this progress (44). Unlike in either the OVCAR-3 cells or MCF-7 cells mentioned above, increased phosphorylation of EGFR and ERK were observed in the BDE-47-treated EC cells. Simultaneously, siRNA for ERα and GPR30 reduced phosphorylated EGFR and ERK expression levels. Moreover, BDE-47-induced estrogen-like effect was partially impaired in cells treated with either an EGFR inhibitor (erlotinib) or an ERK inhibitor (PD98059). The present study is the first to investigate the cross talk between EGFR/ERK and ERα/GPR30 underlined the stimulation of EC cells by BDE-47. GPR30/ERα and EGFR/ERK seem to be the general pathways mediating the impact of BDE-47 on the development of EC.

Previous studies indicated that cadmium, a metalloestrogen, influenced the 5-Fluorouracil cytotoxic effects on breast cancer cells (27, 45). Both clinical observations and experimental studies have suggested that steroid hormones and their receptors affect the therapeutic efficacy of antineoplastic drugs (46). It was demonstrated that ERα/17β-estradiol attenuates therapeutic efficacy of paclitaxel on breast xenograft tumors (47). Another *in vitro* study showed that the increase in ERα expression in ERα-negative Bcap37 breast cancer cells significantly increased their chemoresistance, whereas ERα activation by 17β-estradiol increased the sensitivity of natural ERα-positive T47D breast cancer cells to chemotherapeutic agents (48). Considering the upregulation of ERα and/or GPR30 in BDE-47-treated EC cell lines, we speculate that estrogen signaling pathway is involved in the chemoresistance mechanism. In the present study, the resistance to paclitaxel and DDP induced by BDE-47 was enhanced in both Ishikawa and HEC-1B cells, which suggests that not only the ER alpha status but also other molecular mechanism influence on observed results. Interestingly, up-regulating GPR30 was also found in high-risk endometrial cancer patients with lower survival rates (9) and in tamoxifen-resistant breast cancer cells through the EGFR/ERK transduction pathway (49). More exhaustive and systematic studies are essential to reach deeper understandings on the cross talk between ERα/GPR30 and EGFR/ERK signaling pathway involved in BDE-47 induced chemoresistance as well as the chemoresistance differences in HEC-1-B-DBE-47 and Ishikawa-BDE-47 cells. It suggests that considering the influence of BDE-47 exposure on chemosensitivity, determining the body BDE-47 burden in EC patients might be taken into account when using chemotherapeutic drugs.

The concentration used in this BDE-47 exposure model is based on the dose-response experiment and the data shown previously in other *in vitro* systems (34, 50–52). Recently, Kanaya compared with *in vitro* breast cancer cell culture treatment at dosage of 10 μM to the published human serum/tissue concentrations of PBDEs (BDE-47 included), and concluded that the maximum concentrations observed in human serum and tissue concentrations were ~1,300 and 350 times lower than 10 μM, respectively (32). Therefore, future study will be needed to advance understanding the effect of environmentally relevant low-dose PBDEs on the progression and chemoresistance of EC cells *in vitro* and *in vivo*, which will help to answer the critical question whether real-life BDE-47 exposure could be a risk of human EC and resistance factor for chemotherapy.

In summary, this preliminary study shows BDE-47 promotes cell growth, migration and even chemoresistance of EC cells both *in vivo* and *in vitro*. We speculate that this progress, at least in part, mimicks the effects of estrogen. The cross talk between ERα/GPR30 and the EGFR/ERK signaling pathway might be a crucial mechanism underlying the impact of BDE-47 on EC cell plasticity and chemoresistance.

## Data Availability Statement

The raw data supporting the conclusions of this manuscript will be made available by the authors, without undue reservation, to any qualified researcher.

## Ethics Statement

The animal study was reviewed and approved by Medical Animal Care & Welfare Committee of Shantou University Medical College.

## Author Contributions

FZ performed the statistical analyses and drafted the manuscript. JC designed and performed the experiments. YH and LP helped to draft and revise the manuscript. XL helped to revise this manuscript. LP and LZ conceived of the study and supervised the work. All authors read and approved the final manuscript.

### Conflict of Interest

The authors declare that the research was conducted in the absence of any commercial or financial relationships that could be construed as a potential conflict of interest.
